# Regulation of Dendritic Filopodial Interactions by ZO-1 and Implications for Dendrite Morphogenesis

**DOI:** 10.1371/journal.pone.0076201

**Published:** 2013-10-02

**Authors:** Ryouhei Komaki, Hideru Togashi, Yoshimi Takai

**Affiliations:** 1 Division of Molecular and Cellular Biology, Department of Biochemistry and Molecular Biology, Kobe University Graduate School of Medicine, Kobe, Japan; 2 Division of Pathogenetic Signaling, Department of Biochemistry and Molecular Biology, Kobe University Graduate School of Medicine, Kobe, Japan; 3 CREST, Japan Science and Technology Agency, Kobe, Japan; University of Nebraska Medical Center, United States of America

## Abstract

Neuronal dendrites dynamically protrude many fine filopodia in the early stages of neuronal development and gradually establish complex structures. The importance of the dendritic filopodia in the formation of axo-dendritic connections is established, but their role in dendrite morphogenesis remains unknown. Using time-lapse imaging of cultured rat hippocampal neurons, we revealed here that many filopodia dynamically protruded from dendrites and transiently interacted with each other to form dendritic filopodia-filopodia contacts in the early stages of neuronal development. The MAGUK family member, Zonula Occludens-1 (ZO-1), which is known to be associated with the nectin and cadherin cell adhesion systems, was concentrated at these dendritic filopodia-filopodia contact sites and also at the tips of free dendritic filopodia. Overexpression of ZO-1 increased the formation of dendritic filopodia and their interactions, and induced abnormal dendrite morphology. Conversely, knockdown of ZO-1 decreased the formation of dendritic filopodia and their interactions, and induced abnormal dendrite morphology which was different from that induced by the overexpression of ZO-1. The components of the nectin and cadherin systems were co-localized with ZO-1 at the dendritic filopodia-filopodia contact sites, but not at the tips of free dendritic filopodia. Overexpression of ZO-1 increased the accumulation of these cell adhesive components at the dendritic filopodia-filopodia contact sites and stabilized their interactions, whereas knockdown of ZO-1 reduced their accumulation at the dendritic filopodia-filopodia contact sites. These results indicate that ZO-1 regulates dendritic filopodial dynamics, which is implicated in dendrite morphogenesis cooperatively with the nectin and cadherin systems in cultured neurons.

## Introduction

Developing neurons elongate an axon that attaches to the dendrites of other neurons to form synapses and establish neuronal networks. Dendrites are branched and show complex structures with arbors of different sizes and shapes depending on the type of neurons [1,2]. These dendritic arborization patterns affect the number and pattern of synaptic inputs and the function of brain circuits [3]. Many mechanisms have been proposed to be involved in dendritic arborization [4-6]. Transcriptional regulators, cytoskeletal regulators, motors, cell surface receptors, cell adhesion molecules (CAMs), and extracellular signaling molecules secreted from cells have been shown to regulate dendrite growth and morphology. Of these mechanisms, cytoskeletal regulators such as the Cdc42, Rac and Rho small G proteins regulate dendrite growth and morphology through the reorganization of the actin cytoskeleton [7]. CAMs, such as the Down’s syndrome cell adhesion molecule and proto-cadherins, are implicated in dendrite self-avoidance [8-10]. However, how dendritic arbors take shape is still largely unknown.

When hippocampal neurons are cultured, many processes protrude from the cell body with one becoming an axon, whereas the others become dendrites [11]. Extending axons and dendrites have growth cones at their growing tips, and many filopodia protrude from these growth cones [12,13]. Filopodia are also observed on the shafts of dendrites and axons. When dendritic filopodia interact with axons, some of them are transformed to spines and finally form synapses, whereas the others fail to form synapses [13]. Dendritic filopodia are considered to increase the frequency of meeting with axons, but other functions of these dendritic filopodia remain unknown.

Filopodia are protruded by the polymerization of actin filaments (F-actin), whereas they are retracted by the depolymerization of F-actin [14]. These F-actin dynamics are regulated by Cdc42, whose activity is regulated by extracellular signaling molecules and CAMs [15,16]. Many upstream regulators and downstream effectors of Cdc42 have been identified, one of the upstream regulators being the Cdc42 GEF, Tuba [17]. Zonula Occludens-1 (ZO-1) is an F-actin binding protein [18] that binds to Tuba [19]. ZO-1 is a member of the MAGUK family and was originally identified as a protein that is associated with tight junctions in epithelial cells [20]. ZO-1 comprises a family with three members, ZO-1, ZO-2 and ZO-3, all of which contain three PDZ domains, one SH3 domain and one guanylate kinase domain in this order from the N-terminus, and bind to F-actin [18,21,22]. ZO-1 plays a key role in the formation and maintenance of tight junctions in epithelial and endothelial cells [23].

In addition to Tuba, ZO-1 binds to α-catenin [18] and afadin [24]. α-Catenin is associated with the CAMs, cadherins, through β-catenin, and afadin is directly associated with the CAMs, nectins [16,25,26]. Cadherins comprise a superfamily consisting of over one hundred members, and N-cadherin is a member of the classic cadherin family and is expressed in neurons [27]. Classic cadherins such as N-cadherin, E-cadherin and VE-cadherin only interact homophilically *in trans* with each other to induce cell–cell adhesion. Nectins comprise a family of four members (nectin-1, nectin-2, nectin-3 and nectin-4) [16,25]. Nectins interact homophilically and heterophilically *in trans* with each other to induce cell–cell adhesion. Cell–cell adhesion is initiated by the nectin-based cell–cell adhesion, which recruits the cadherin-catenin complex to the nectin-based cell–cell adhesion sites to stabilize the cell–cell adhesion, and eventually results in the formation of adherens junctions in a variety of cells including fibroblasts, epithelial cells and endothelial cells. The trans-interactions of nectins induce the activation of Cdc42, which is also involved in the recruitment of the cadherin-catenin complex to the nectin-based cell–cell adhesion sites through reorganization of the actin cytoskeleton. At the mossy fiber-CA3 pyramidal cell synapses in the stratum lucidum of the mouse hippocampus, nectin-1 and nectin-3 are asymmetrically localized on the presynaptic and postsynaptic sides, respectively, whereas afadin, N-cadherin, β-catenin and αN-catenin are symmetrically localized on both sides [28]. This asymmetric localization of nectin-1 on axons and nectin-3 on dendrites and their heterophilic trans-interactions are at least partly involved in the selective interaction of axons and dendrites [29], because the trans-interaction between nectin-1 and nectin-3 is the strongest of the various combinations of trans-interactions between the nectin family members [16]. We previously showed that ZO-1 is co-localized with nectins and afadin at the mossy fiber-CA3 pyramidal cell synapses of the stratum lucidum in the mouse hippocampus and that this localization of ZO-1 is dependent on nectins and afadin [30]. However, the involvement of ZO-1 or nectins in the regulation of neuronal morphogenesis remains unknown.

We investigated here the role of dendritic filopodia in cultured rat hippocampal neurons. We found that most dendritic filopodia that did not interact with axons dynamically protruded and transiently interacted with each other to form dendritic filopodia-filopodia contacts, and that ZO-1 plays a role in dendrite morphogenesis cooperatively with the nectin and cadherin systems by regulating these dynamic behaviors of dendritic filopodia in cultured neurons.

## Results

### Dynamic protrusion of and transient interactions between dendritic filopodia in cultured hippocampal neurons

**Figure 1:**
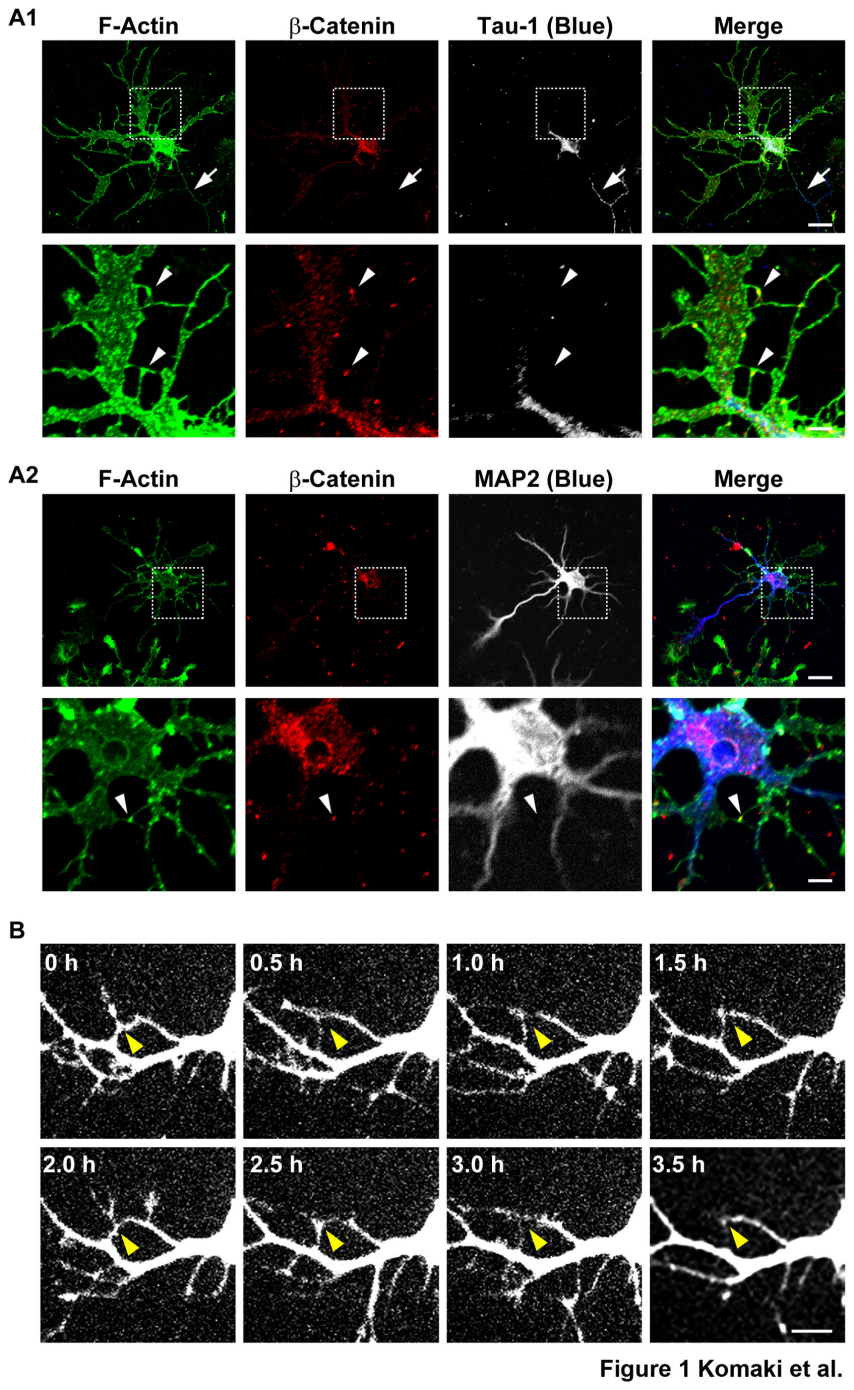
Transient interactions between dendritic filopodia in cultured hippocampal neurons. (**A**) Triple immunostaining for F-actin, β-catenin and either Tau-1 or MAP2 in cultured hippocampal neurons. Cultured neurons on 5 DIV were triple-stained for F-actin, β-catenin and either Tau-1 or MAP2. (A1) F-actin, β-catenin and Tau-1; (A2) F-actin, β-catenin and MAP2. **Upper** **rows**, low magnification images; **lower** **rows**, high magnification images of the boxed areas in the upper rows. **Bars, upper** **rows** 10 µm; **lower** **rows** 2.5 µm. Dendrites and axons were identified by the signals for MAP2 and Tau-1. Arrows indicate axons and arrowheads indicate dendritic filopodia-filopodia contact sites, which were identified by the signal for β-catenin. (**B**) Time-lapse imaging of EGFP-expressing neurons. Time-lapse images of EGFP-expressing neurons on 4 DIV were acquired every 30 min. **Bar**, 5 µm. Arrowheads indicate dendritic filopodia-filopodia contact sites. We identified neurites to be dendrites in (**B**) as follows: we identified MAP2-poitive and Tau-1-negative neurites to be dendrites and MAP2-negative and Tau-1-positive neurites to be axons; in cultured neurons on 4 DIV, axons were generally longer than dendrites; and the diameters of axons, which were 10-µm apart from the cell body, became smaller than those of dendrites at the same distance. We identified neurites to be dendrites by these characteristic morphologies.

Rat hippocampal neurons were cultured for various days in vitro (DIV). On 5 DIV, many neurites bearing growth cones and filopodia protruded from neurons, as estimated by the immunofluorescence signal for F-actin (Figure 1, A1 and A2). The longest neurite was identified to be an axon by the signal for the axonal marker, Tau-1 (Figure **1A1** **, arrows**), and the others were identified to be dendrites by the signal for the dendritic marker, MAP2. Many filopodia protruded from dendrites. Some of these dendritic filopodia interacted with axons (data not shown). However, many of the dendritic filopodia that did not meet with axons interacted with each other, and the signal for β-catenin was concentrated at the contact sites (Figure 1, A1 and A2 **, arrowheads**). These dendritic filopodia-filopodia contacts gradually decreased in number and finally disappeared on 14 DIV (Figure **S1A**). In contrast, the dendritic filopodia-axons contacts were still observed on 14 DIV (Figure **S1A** **, arrowheads**).

We then performed time-lapse analysis of neurons transfected with an EGFP vector from 3 to 7 DIV. Some primary dendrites and their branches dynamically protruded and retracted from their cell body and the shafts of the primary dendrites, respectively (Video **S1**). Filopodia dynamically protruded from dendrites and transiently interacted with each other. These filopodia protruded repetitively from different sites on dendrites. Most of these interactions continued at least for 1 h, followed by detachment and retraction, but sometimes continued for 1 to 24 h. Time-lapse images of the dynamic behaviors of dendritic filopodia protruding from dendrites, which were taken every 0.5 h, are shown (Figure **1B**). Taken together, these results indicate that most of the dendritic filopodia that do not interact with axons dynamically and repetitively protrude and transiently interact with each other to form dendritic filopodia-filopodia contacts in the early stages of neuronal development.

### Localization of ZO-1 at the tips of free dendritic filopodia and the dendritic filopodia-filopodia contact sites

**Figure 2:**
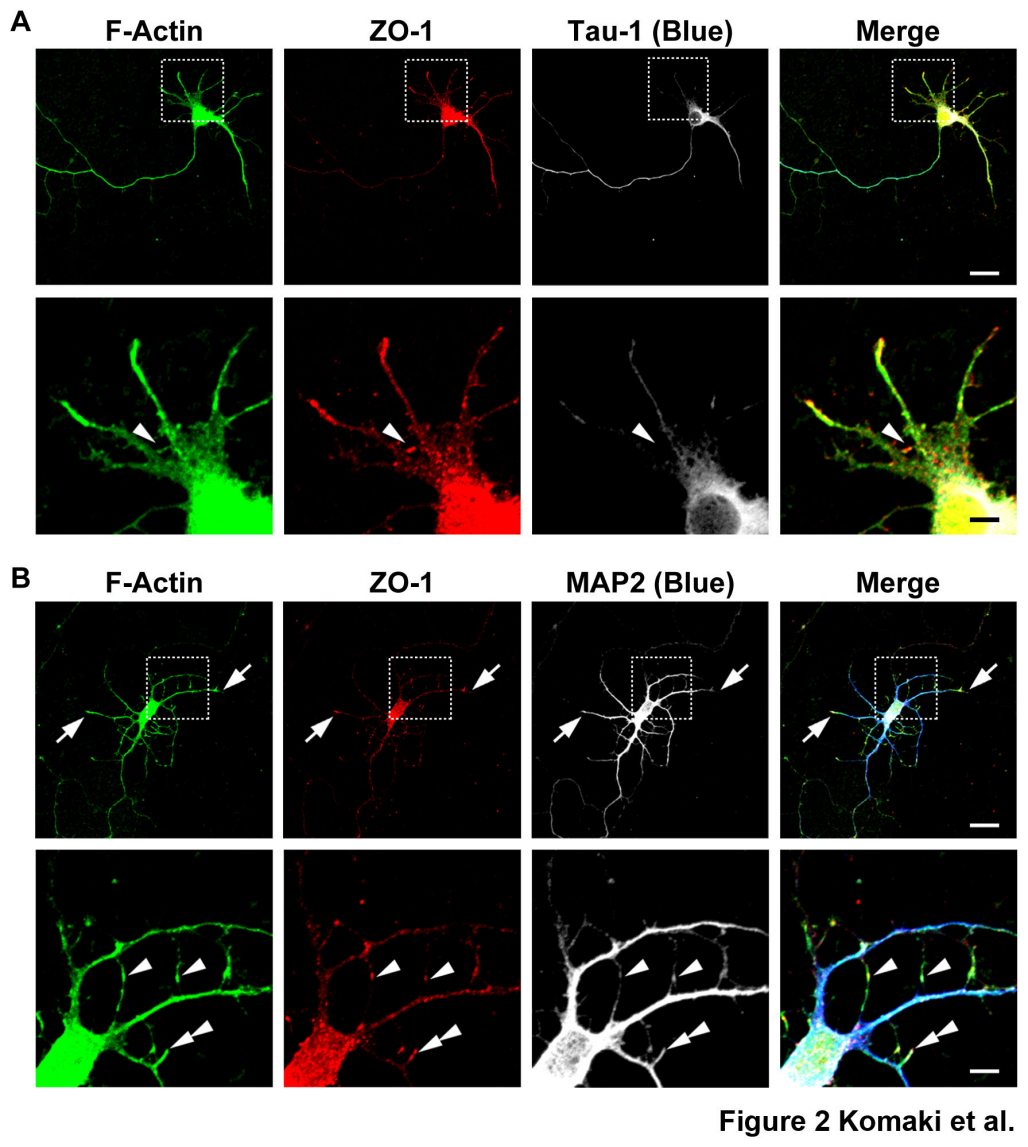
Localization of ZO-1 at the tips of free dendritic filopodia and filopodia-filopodia contact sites in cultured hippocampal neurons. Triple immunostaining for F-actin, ZO-1 and either Tau-1 or MAP2 in cultured hippocampal neurons. Cultured neurons on 5 DIV were triple-stained for F-actin, ZO-1 and either Tau-1 or MAP2. (**A**) F-actin, ZO-1 and Tau-1; (**B**) F-actin, ZO-1 and MAP2. **Upper** **rows**, low magnification images; **lower** **rows**, high magnification images of the boxed areas in the upper rows. **Bars**, **upper** **rows** 10 µm; **lower** **rows** 2.5 µm. Dendrites and axons were identified by the signals for MAP2 and Tau-1. Arrows indicate the growth cones of dendrites. Double arrowheads and arrowheads indicate the tips of free dendritic filopodia and dendritic filopodia-filopodia contact sites, respectively. It is noted that multiple Tau-1-positive neurites were observed in the neurons shown in (**A**) as described previously [34]. One of these multiple Tau-1-positive neurites is specified as an axon in the later stages of neuronal development [34].

We then investigated a possible involvement of ZO-1 in these dynamic behaviors of dendritic filopodia. We first examined the distribution of ZO-1 in cultured hippocampal neurons on 5 DIV. In this stage, the immunofluorescence signal for ZO-1 was observed diffusely throughout the cell body, dendrites and axons (Figure **2** **, A and B**), but was observed at the growth cones of dendrites (Figure **2B** **, arrows**) and about 40% of the tips of dendritic filopodia (Figure **2B** **, double arrowheads**). The signal for ZO-1 was also concentrated at the dendritic filopodia-filopodia contact sites (Figure **2** **, A and B, arrowheads**). It was observed at almost all of the contact sites. The dendritic filopodia-filopodia contacts were identified by the concentration of the signals for the components of the nectin and cadherin systems (**see below**). The signal for ZO-1 was also markedly concentrated at the dendritic filopodia-axons contact sites (data not shown). It was observed at almost all of these contact sites. The signal for ZO-1 at the dendritic filopodia-filopodia contact sites gradually decreased and disappeared on 14 DIV (Figure **S1B**). In contrast, the signal for ZO-1 at the dendritic filopodia-axons contact sites persisted and was still observed on 14 DIV (Figure **S1B** **, arrowheads**). These results indicate that ZO-1 is localized at the tips of free dendritic filopodia and the dendritic filopodia-filopodia contact sites in the early stages of neuronal development.

### Enhancement of dendritic filopodia protrusion, stabilization of their interactions, and changes in dendrite morphology in ZO-1-overexpressing neurons

**Figure 3:**
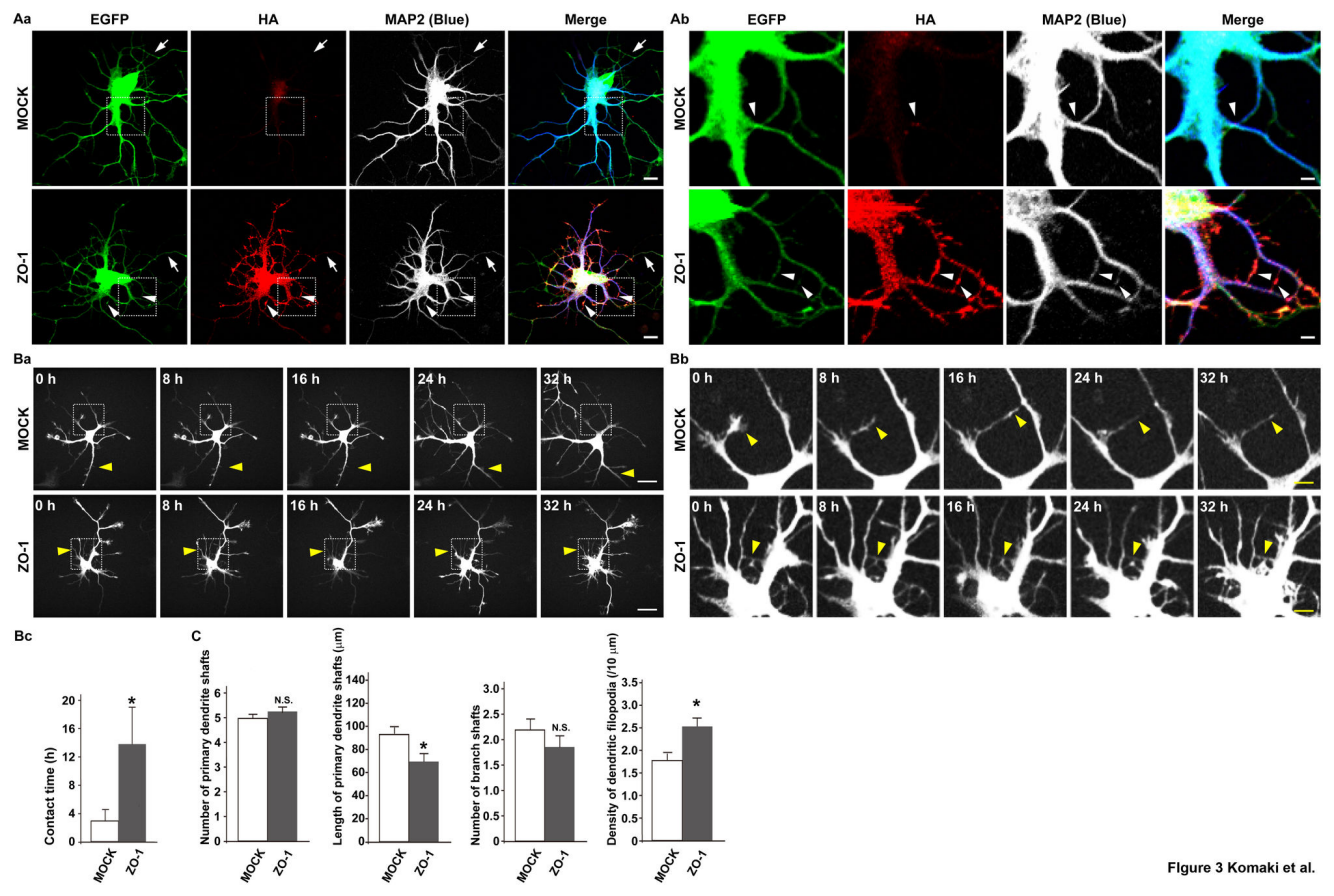
Enhancement of dendritic filopodia protrusion and changes in dendrite morphology in ZO-1-overexpressing neurons. (**A**) Dendrite morphologies of the control and HA-tagged ZO-1-overexpressing neurons. Cultured neurons were transfected with EGFP and either the empty vector (MOCK) or the HA-tagged ZO-1 vector (ZO-1) on 0 DIV and triple-stained for EGFP, HA and MAP2 on 7 DIV. (Aa) low magnification images; (Ab) high magnification images of the boxed areas in (Aa). **Upper** **rows**, control neurons**, lower** **rows**, HA-tagged ZO-1-overexpressing neurons. **Bars**, (Aa) 10 µm; (Ab) 2.5 µm. Arrows and arrowheads indicate axons and dendritic filopodia-filopodia contact sites, respectively. (**B**) Time-lapse imaging of the control and HA-tagged ZO-1-overexpressing neurons. Time-lapse images of the neurons expressing EGFP and either the empty vector or the HA-tagged ZO-1 vector were acquired every 8 h from 5 to 6 DIV. (Ba) low magnification images; (Bb) high magnification images of the boxed areas in (Ba). **Upper** **rows**, neurons expressing EGFP and the empty vector; **lower** **rows**, neurons expressing EGFP and the HA-tagged ZO-1 vector. **Bars**, (Ba) 10 µm; (Bb) 2.5 µm. Arrowheads in (Ba) and (Bb) indicate dendrites and dendritic filopodia-filopdoia contact sites, respectively. (Bc) Quantitation of the dendritic filopodia-filopodia contact time. The data are presented as mean plus SEM (error bars) for each sample (n = 25). *, p value < 0.05 (Mann-Whitney U test). (**C**) Quantitation of the dendrite morphologies of HA-tagged ZO-1-overexpressing neurons. The average number of the shafts of the primary dendrites, the average length of the shafts of the primary dendrites, the average number of the shafts of the branches of the primary dendrites, and the average density of the dendritic filopodia protruding from the primary dendrites in the neurons transfected with the empty vector or the HA-tagged ZO-1 vector on 0 DIV were measured on 7 DIV. The data are presented as mean plus SEM (error bars) for each sample (n = 20 for the number of the shafts of the primary dendrites (Number of primary dendrite shafts); n = 64 for the length of the shafts of the primary dendrites (Length of primary dendrite shafts) and the number of the shafts of the branches of the primary dendrites (Number of branch shafts); n = 46 for the density of the dendritic filopodia protruding from the primary dendrites (Density of dendritic filopodia). *, p value < 0.05 (Student’s t-test)).

We then examined the effects of ZO-1 overexpression on dendrite morphology in cultured hippocampal neurons on 7 DIV. We transfected the neurons with the EGFP vector and either the empty vector or the HA-tagged ZO-1 vector. In the HA-tagged ZO-1-overexpressing neurons, the distribution pattern of the signal for HA-tagged ZO-1 was similar to that of the signal for endogenous ZO-1 (Figure **3A** **, a and b**). The signal for HA-tagged ZO-1 was observed at all the dendritic filopodia-filopodia contact sites in the ZO-1-overexpressing neurons. However, the overexpression of HA-tagged ZO-1 caused abnormal dendrite morphology: (1) the length of the shafts of the primary dendrites protruding from the cell body was decreased while the number of these dendritic shafts was not significantly changed: the average length was 92.63 ± 5.13 µm in the control neurons, whereas that was 70.37 ± 6.03 µm in the ZO-1-overexpressing neurons; and the average number was 4.9 ± 0.2 in the control neurons, whereas that was 5.27 ± 0.37 in the ZO-1-overexpressing neurons. These values were estimated by the signals for EGFP and MAP2 and are presented as mean ± SEM; (2) the density of dendritic filopodia was increased without a significant change in the number of the shafts of the branches of the primary dendrites: the average density was 1.79 ± 0.14 per 10 µm in the control neurons, whereas that was 2.55 ± 0.17 per 10 µm in the ZO-1-overexpressing neurons; and the average number was 2.20 ± 0.20 in the control neurons, whereas that was 1.87 ± 0.24 in the ZO-1-overexpressing neurons. These values were estimated by the signals for EGFP and MAP2 and are presented as mean ± SEM; and (3) primary dendrites protruded radially from the cell body, similar to the control neurons, but the dendrite morphology became more complex than that in the control neurons (Figure **3A** **, a and b, and** Figure **3C**). The expression of HA-tagged ZO-1 in the cultured neurons transfected with the EGFP vector and either the empty vector or the HA-tagged ZO-1 vector was confirmed by Western blotting using the anti-HA monoclonal antibody (mAb) (Figure **S2A**).

We then performed time-lapse analysis of the neurons transfected with the EGFP vector and either the empty vector or the HA-tagged ZO-1 vector and cultured for 5 to 6 DIV. In the HA-tagged ZO-1-overexpressing neurons, the dynamic movements of dendrites and dendritic filopodia seen in the control neurons (Video **S2**) were slowed down (Video **S3**): the average dendritic filopodia-filopodia contact time was 2.87 ± 1.70 h in the control neurons, whereas that was 13.72 ± 4.83 h in the ZO-1-overexpressing neurons (**Figure 3Bc**). These data are presented as mean ± SEM. Thus, the dendritic filopodia-filopodia contact time was increased in the ZO-1-overexpressing neurons as compared to that in the control neurons. The morphological changes shown in Figure **3A** **, a and b**, were all confirmed in these videos. Time-lapse images of the dynamic behaviors of dendritic filopodia, which were taken every 8 h, are shown (Figure **3B** **, a and b**). It was confirmed by the immunostaining of HA that HA-tagged ZO-1 was overexpressed in the neurons shown in Figure **3B** **, a and b** (data not shown). These results indicate that the overexpression of ZO-1 enhances the protrusion of dendritic filopodia, stabilizes their interactions, and changes dendrite morphology.

### Reduction of dendritic filopodia protrusion and changes in dendrite morphology in ZO-1-knockdown neurons

**Figure 4:**
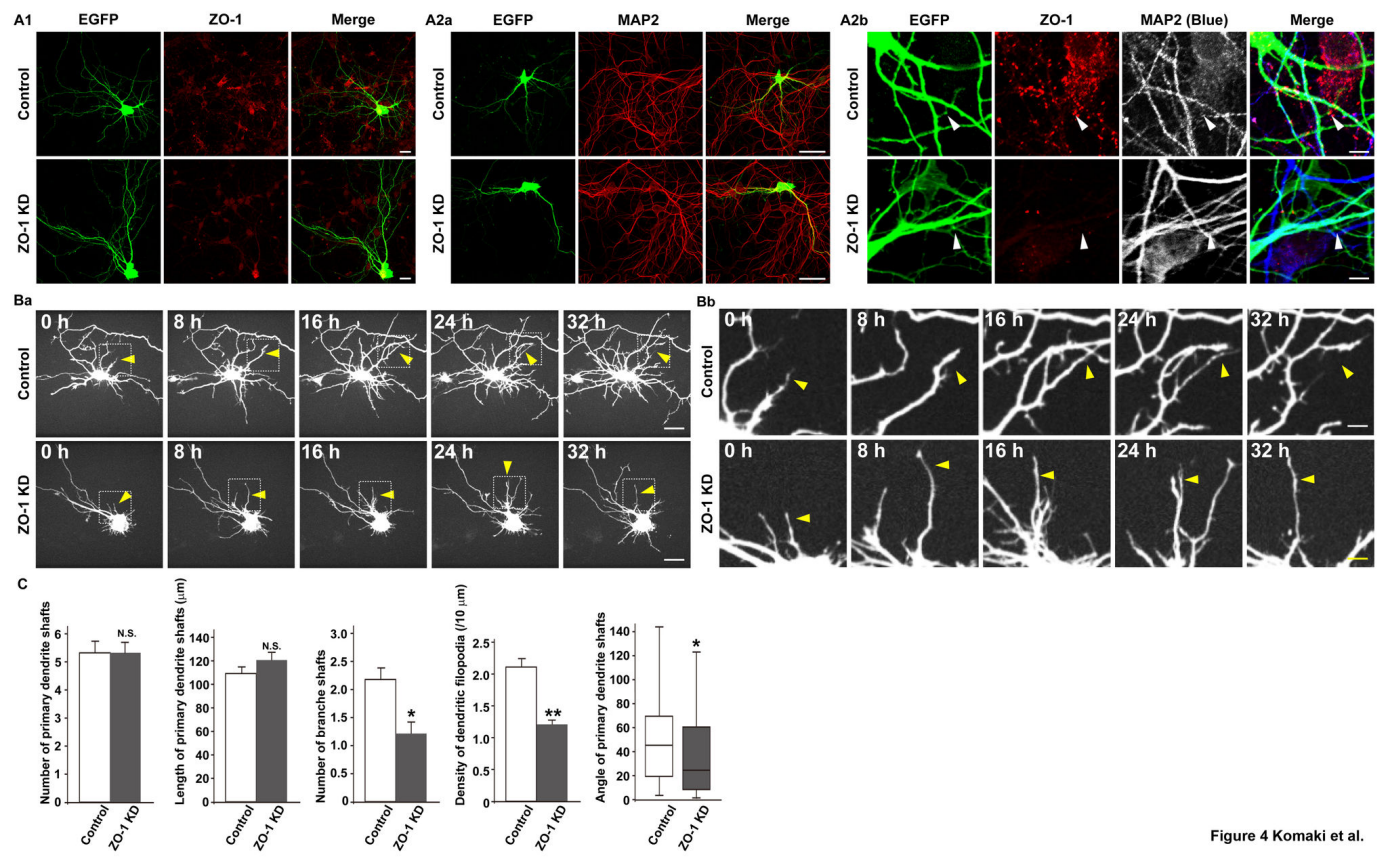
Reduction of dendritic filopodia protrusion and changes in dendrite morphology in ZO-1-knockdown neurons. (**A**) Dendrite morphologies of the control and ZO-1-knockdown (ZO-1 KD) neurons. Cultured neurons were transfected with the control siRNA or the ZO-1 siRNA on 3 DIV, then transfected with EGFP on 5 DIV, and double-immunostained for EGFP and either ZO-1 or MAP2 on 7 DIV. (A1) EGFP and ZO-1; (A2a) EGFP and MAP2; (A2b) EGFP, ZO-1 and MAP2. **Upper** **rows**, control neurons; **lower** **rows**, ZO-1-knockdown neurons. **Bars**, (A1) 10 µm; (A2a) 20 µm; (A2b) 2.5 µm. (**B**) Time-lapse imaging of the control and ZO-1-knockdown (ZO-1 KD) neurons. Time-lapse images of the neurons expressing EGFP and either the control siRNA or the ZO-1 siRNA were acquired every 8 h from 5 to 6 DIV. (Ba) low magnification images; (Bb) high magnification images of the boxed areas in (Ba). **Upper** **rows**, neurons expressing EGFP and the control siRNA; **lower** **rows**, neurons expressing EGFP and the ZO-1 siRNA. **Bars**, (Ba) 10 µm; (Bb) 2.5 µm. Arrowheads indicate dendrites. (**C**) Quantitation of the dendrite morphologies of ZO-1-knockdown neurons. The average number of the shafts of the primary dendrites, the average length of the shafts of the primary dendrites, the average number of the shafts of the branches of the primary dendrites, the average density of the dendritic filopodia protruding from the primary dendrites and the median angle of the shafts of the primary dendrites in the neurons transfected with the control siRNA or the ZO-1 siRNA on 3 DIV were measured on 7 DIV. The data are presented as mean plus SEM (error bars) for each sample (n = 21 for the number of the shafts of the primary dendrites (Number of primary dendrite shafts); n = 60 for the length of the shafts of the primary dendrites (Length of primary dendrite shafts), the number of the shafts of the branches of the primary dendrites (Number of branch shafts) and the angles of the shafts of the primary dendrites (Angle of primary dendrite shafts); n = 33 for the density of the dendritic filopodia protruding from the primary dendrites (Density of dendritic filopodia)). Statistical analyses of the dendrite morphology of the ZO-1-knockdown neurons except for that of the angles of the shafts of each dendrite were performed using Student’s t-test. Statistical analysis of the angles of the shafts of each primary dendrite was performed using the Mann-Whitney U test. *, p value < 0.05; and **, p value < 0.01 (Student’s t-test) and *, p value <0.05 (Mann-Whitney U test).

We next examined the effects of ZO-1 knockdown on dendrite morphology in cultured hippocampal neurons on 7 DIV. We transfected the neurons with the EGFP vector and either the negative control small interfering RNA (siRNA) or the ZO-1 siRNA. In the ZO-1-knockdown neurons, the immunofluorescence signal for ZO-1 was reduced at most places where it was concentrated in the control neurons (Figure **4A1**). The knockdown of ZO-1 caused abnormal dendrite morphology, which was different from those in the control and ZO-1-overexpressing neurons: (1) the length of the shafts of the primary dendrites and the number of these dendritic shafts were not significantly different from those of the control neurons: the average length was 109.69 ± 6.79 µm in the control neurons, whereas that was 121.15 ± 7.74 µm in the ZO-1-knockdown neurons; and the average number was 5.33 ± 0.46 in the control neurons, whereas that was 5.31 ± 0.40 in the ZO-1-knockdown neurons. These values were estimated by the signals for EGFP and MAP2 and are presented as mean ± SEM; (2) the number of the shafts of the branches of the primary dendrites and the density of dendritic filopodia were markedly decreased: the average number was 2.17 ± 0.21 in the control neurons, whereas that was 1.2 ± 0.20 in the ZO-1-knockdown neurons; and the average density was 2.18 ± 0.19 per 10 µm in the control neurons, whereas that was 1.17 ± 0.16 per 10 µm in the ZO-1-knockdown neurons. These values were estimated by the signals for EGFP and MAP2 and are presented as mean ± SEM; (3) the shafts of the primary dendrites extended apparently in parallel and were fasciculated, different from the shafts of the primary dendrites in the control neurons, which extended radially from the cell body. The angles of the shafts of each primary dendrite of the ZO-1-knockdown neurons were decreased; the median angle was 47.13 degree in the control neurons, whereas that was 26.75 degree in the ZO-1-knockdown neurons. These values were estimated by the signals for EGFP and MAP2; and (4) the dendrite morphology was less complex than that in the control neurons (Figure **4A2** **, a and b, and** Figure **4C**). The knockdown of ZO-1 in the neurons transfected with the EGFP vector and either the control siRNA or the ZO-1 siRNA was confirmed by Western blotting using the anti-ZO-1 mAb (Figure **S2B**)**.**

To exclude the possibility of the non-specific action of the ZO-1 siRNA, we performed the rescue experiment by transfecting the ZO-1-knockdown neurons with an siRNA-resistant HA-tagged ZO-1 vector. The transfection of the siRNA-resistant HA-tagged ZO-1 vector significantly increased the number of dendritic filopodia, but did not significantly increase the numbers of the shafts of primary dendrites and their branches or the length of the shafts of primary dendrites (Figure **S3** **, A and B**). In addition, the transfection of the siRNA-resistant HA-tagged ZO-1 vector decreased the fasciculation of the shafts of primary dendrites, increased the angles of the shafts of each primary dendrite, and restored the complex dendrite morphology. The insufficient restoration of these phenotypes might be just due to the insufficient expression of HA-tagged ZO-1. In contrast, the transfection of the empty vector did not affect any phenotype observed in the ZO-1-knockdown neurons. These results indicate that the phenotypes of the ZO-1-knockdown neurons were not simply caused by the non-specific action of the ZO-1 siRNA.

We then performed time-lapse analysis of the neurons transfected with the EGFP vector and either the control siRNA or the ZO-1 siRNA on 3 DIV and cultured for 5 to 6 DIV. In the ZO-1-knockdown neurons, the dynamic movements of dendrites, including the shafts of primary dendrites, their branches and dendritic filopodia, were different from those of the control neurons (Video **S4** and Video **S5**). Particularly, extension of the shafts of primary dendrites from the cell body in parallel in the same direction was obviously observed. It was practically difficult to measure the dendritic filopodia-filopodia contact time in the ZO-1-knockdown neurons, because the number of the dendritic filopodia-filopodia contacts was markedly reduced. The morphological changes shown in Figure **4A2** **, a and b**, were all confirmed in these videos. Representative images of the dynamic behaviors of dendritic filopodia, which were taken every 8 h, are shown (Figure **4B** **, a and b**). It was confirmed by the immunostaining of ZO-1 that ZO-1 was knocked down in the neurons shown in Figure **4B** **, a and b** (data not shown). These results indicate that the knockdown of ZO-1 decreases the protrusion of dendritic filopodia and changes dendrite morphology which was different from that observed in the ZO-overexpressing neurons.

### Co-localization of the components of the nectin and cadherin systems with ZO-1 at the dendritic filopodia-filopodia contact sites

**Figure 5:**
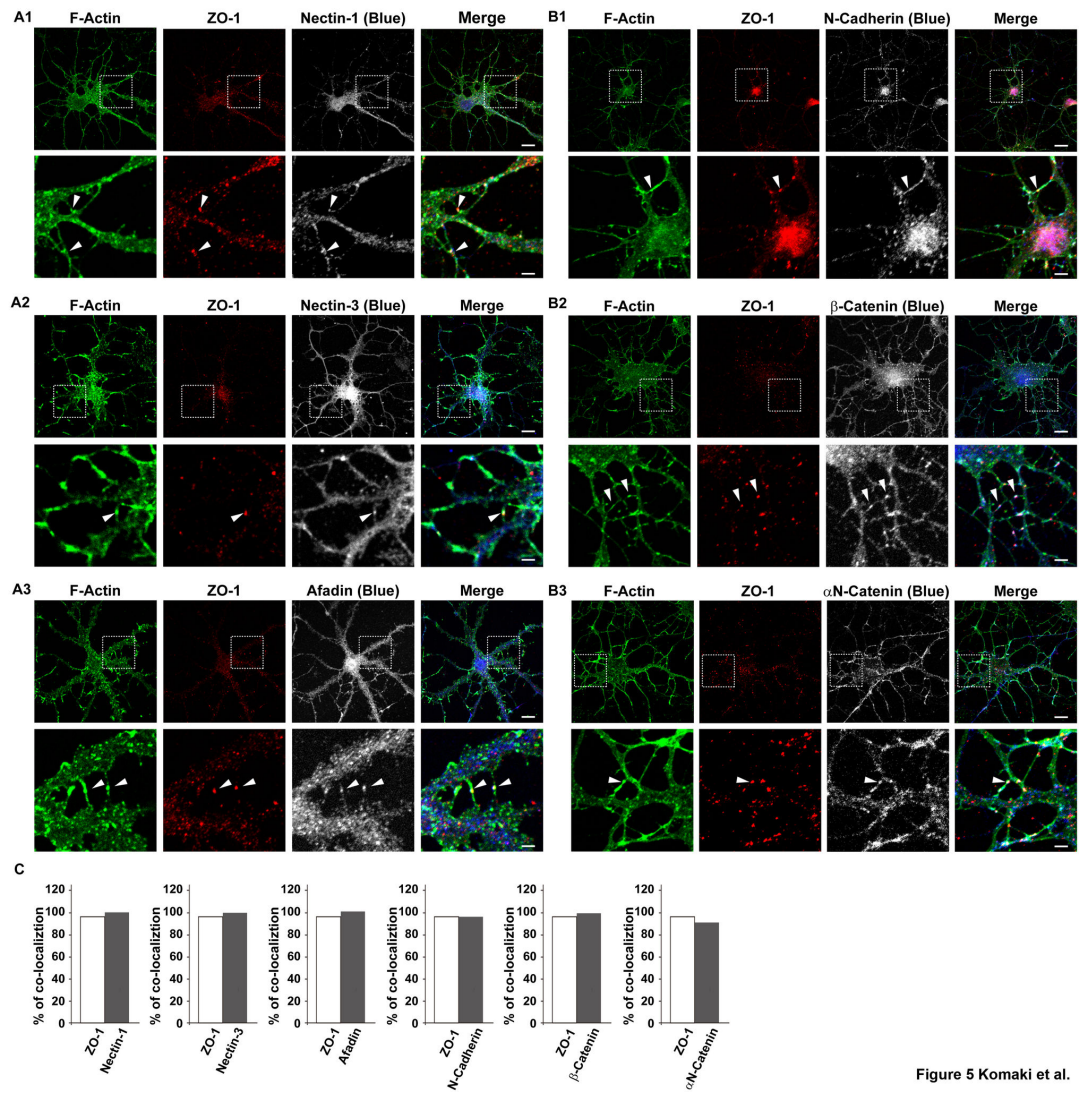
Co-localization of the components of the nectin and cadherin systems with ZO-1 in cultured hippocampal neurons. Cultured hippocampal neurons on 7 DIV were triple-stained for F-actin, ZO-1 and one of nectin-1, nectin-3, afadin, N-cadherin, β-catenin or αN-catenin. (A1) F-actin, ZO-1 and nectin-1; (A2) F-actin, ZO-1 and nectin-3; (A3) F-actin, ZO-1 and afadin; (B1) F-actin, ZO-1 and N-cadherin; (B2) F-actin, ZO-1 and β-catenin; (B3) F-actin, ZO-1 and αN-catenin. **Upper** **rows**, low magnification images; **lower** **rows**, high magnification images of the boxed areas in the upper rows. **Bars**, **upper** **rows** 10 µm; **lower** **rows** 2.5 µm. Arrowheads indicate dendritic filopodia-filopodia contact sites. (**C**) Quantitation of the co-localization of the components of the nectin and cadherin systems with ZO-1 in control neurons. In each experiment, 30 punctate immunofluorescence signals for F-actin at dendritic filopodia-filopodia contact sites were randomly chosen and the percentage of the punctate signal for ZO-1, nectin-1, nectin-3, afadin, N-cadherin, β-catenin or αN-catenin, which was co-localized with that for F-actin (% of co-localization), was counted.

To explore the mechanisms by which ZO-1 regulates dendrite morphology, we examined the effects of ZO-1 on the localizations of the components of the nectin and cadherin systems. We first compared the distribution patterns of nectin-1, nectin-3, afadin, N-cadherin, β-catenin and αN-catenin with that of ZO-1 in cultured hippocampal neurons on 7 DIV. The faint immunofluorescence signals for nectin-1, nectin-3, afadin, N-cadherin, β-catenin and αN-catenin were detected diffusely throughout the cell body, neurites and dendritic filopodia (Figure **5, A1-A3** **and B1-B3**). None of these signals except that for ZO-1 was concentrated at the tips of free dendritic filopodia. At the dendritic filopodia-filopida contact sites, the signals for nectin-1, nectin-3, afadin, N-cadherin, β-catenin and αN-catenin were all concentrated and co-localized with the signal for ZO-1 (Figure **5, A1-A3** **and B1-B3, arrowheads, and** Figure **5C**). All of these signals appeared punctate. On 14 DIV, the filopodia-filopodia contact sites mostly disappeared and all of these signals were not significantly observed at any location where they were observed in the neurons on 7 DIV (Figure **S1A**). At the dendritic filopodia-axons contact sites, the signals for nectin-1, nectin-3, afadin, N-cadherin, β-catenin and αN-catenin were all concentrated and co-localized with the signal for ZO-1 and appeared punctate on 7 DIV (data not shown). On 14 DIV, these signals as well as the signal for ZO-1 at these contact sites were still observed (data not shown). These results indicate that the components of both the nectin and cadherin systems are all concentrated and co-localized with ZO-1 at the dendritic filopodia-filopodia contact sites.

### Increased accumulation of the components of the nectin and cadherin systems at the dendritic filopodia-filopodia contact sites in ZO-1-overexpressing neurons

**Figure 6:**
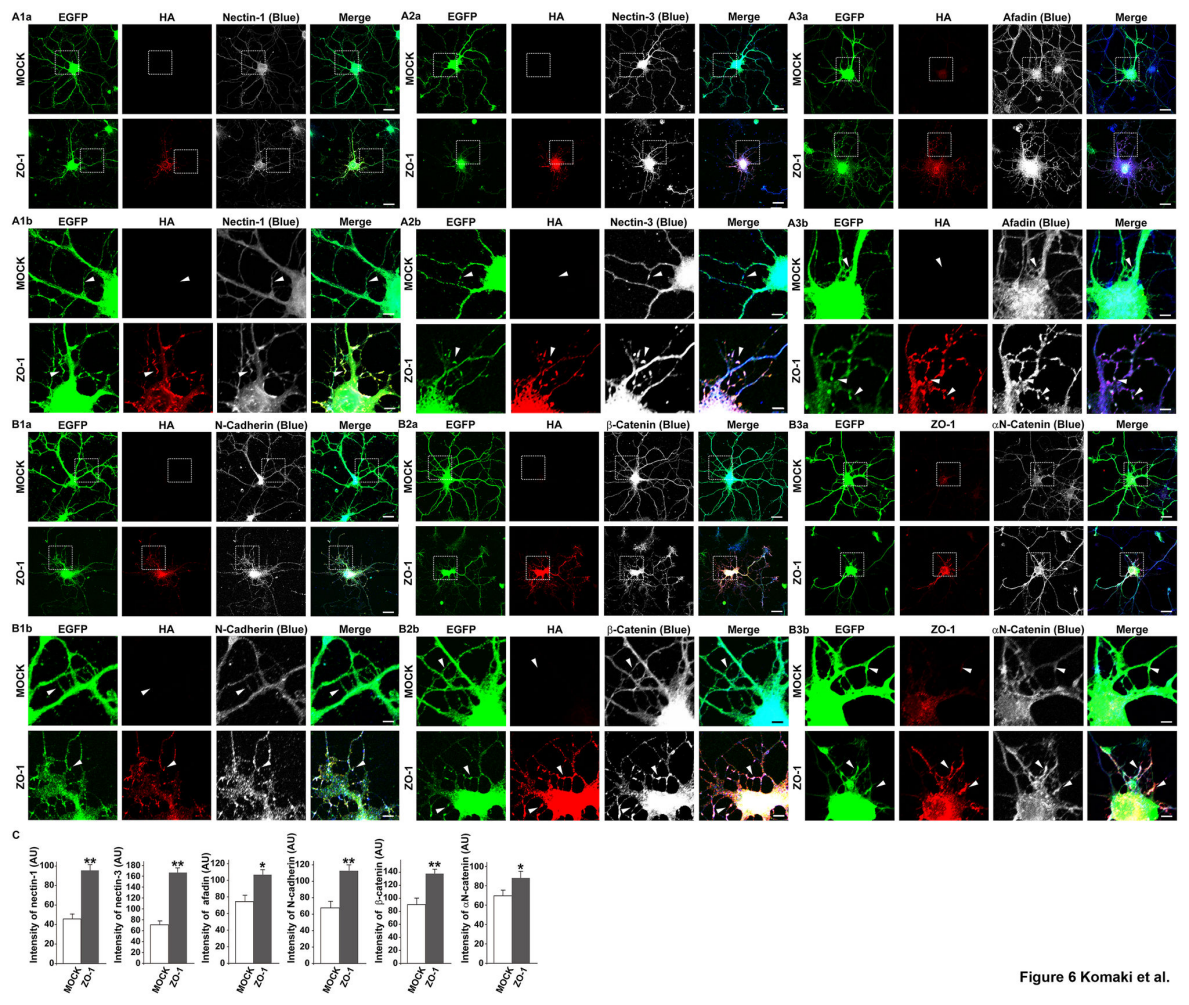
Increased accumulation of the components of the nectin and cadherin systems at the dendritic filopodia-filopodia contact sites in ZO-1-overexpressing neurons. Cultured ZO-1-overexpressing hippocampal neurons on 7 DIV were prepared as described in the legend to Figure **3A** and triple-stained for HA, EGFP and one of nectin-1, nectin-3, afadin, N-cadherin, β-catenin or αN-catenin. (A1) EGFP, HA and nectin-1; (A2) EGFP, HA and nectin-3; (A3) EGFP, HA and afadin; (B1) EGFP, HA and N-cadherin; (B2) EGFP, HA and β-catenin; (B3) EGFP, ZO-1 and αN-catenin. (**a**) low magnification images; (**b**) high magnification images of the boxed areas in (**a**). **Upper** **rows**, control neurons; **lower** **rows**, HA-tagged ZO-1-overexpressing neurons. **Bars**, **upper** **rows** 10 µm; **lower** **rows** 2.5 µm. Arrowheads indicate dendritic filopodia-filopodia contact sites. (**C**) Quantitation of the co-localization of the components of the nectin and cadherin systems with ZO-1 in ZO-1-overexpressing neurons. The data are presented as mean plus SEM (error bars) for each sample (n = 40 for nectin-1, afadin and N-cadherin; n= 50 for nectin-3 and β-catenin; n = 60 for αN-catenin). *, p value < 0.05; and **, p value < 0.01 (Student’s t-test) (AU) arbitrary unit.

We then compared the distribution patterns of the components of both the nectin and cadherin systems with that of HA-tagged ZO-1 in the neurons transfected with the EGFP vector and the HA-tagged ZO-1 vector. In the HA-tagged ZO-1-overexpressing neurons, the immunofluorescence signals for nectin-1, nectin-3, afadin, N-cadherin, β-catenin and αN-catenin were all increased at the dendritic filopodia-filopodia contact sites as compared with those in the control neurons, similar to the signal for HA-tagged ZO-1 (Figure **6, A1-A3** **and B1-B3, arrowheads, and** Figure **6C**): the average intensities at the dendritic filopodia-filopodia contact sites in the control neurons and the HA-tagged ZO-1-overexpressing neurons in arbitrary unit were 46.45 ± 5.18 and 96.43 ± 5.73 for nectin-1, 71.9 ± 7.53 and 167.72 ± 8.73 for nectin-3, 74.48 ± 8.55 and 107.32 ± 7.39 for afadin, 67.21 ± 8.50 and 113.23 ± 6.70 for N-cadherin, 89.52 ± 10.08 and 135.3 ± 8.14 for β-catenin, and 67.43 ± 6.57 and 88.14 ± 7.35 for αN-catenin, respectively. These values are presented as mean ± SEM. These results indicate that the overexpression of ZO-1 increases the accumulation of the components of the nectin and cadherin systems at the dendritic filopodia-filopodia contact sites.

### Reduced accumulation of the components of the nectin and cadherin systems at the dendritic filopodia-filopodia contact sites in ZO-1-knockdown neurons

**Figure 7:**
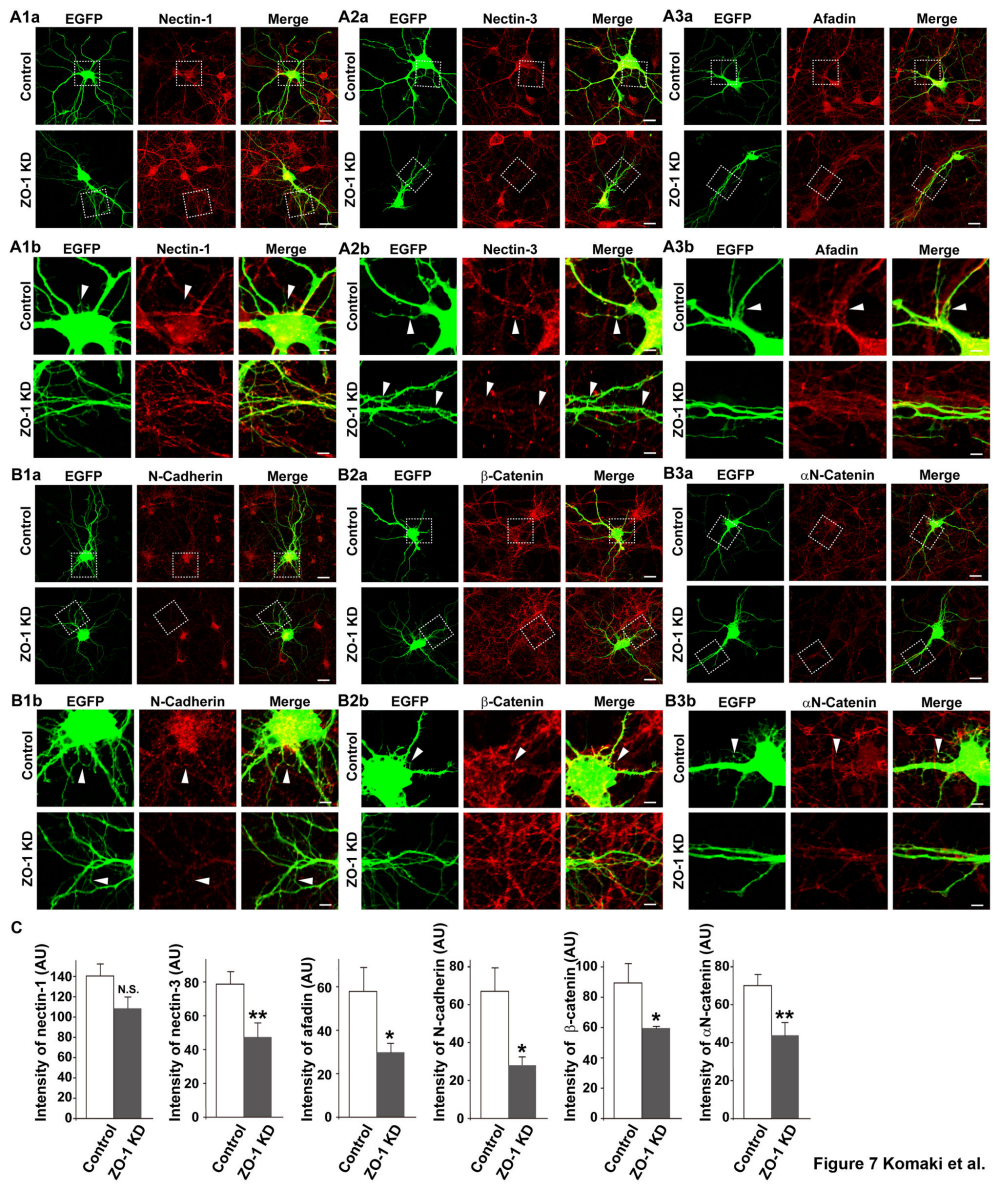
Reduced accumulation of the components of the nectin and cadherin systems at the dendritic filopodia-filopodia contact sites in ZO-1-knockdown neurons. Cultured ZO-1-knockdown hippocampal neurons on 7 DIV were prepared as described in the legend to Figure **4A** and double-stained for EGFP and one of nectin-1, nectin-3, afadin, N-cadherin, β-catenin or αN-catenin. (A1) EGFP and nectin-1; (A2) EGFP and nectin-3; (A3) EGFP and afadin; (B1) EGFP and N-cadherin; (B2) EGFP and β-catenin; (B3) EGFP and αN-catenin; (**a**) low magnification images; (**b**) high magnification images of the boxed areas in (**a**). **Upper** **rows**, control neurons; **lower** **rows**, ZO-1-knockdown neurons. **Bars**, **upper** **rows** 10 µm; **lower** **rows** 2.5 µm. Arrowheads indicate dendritic filopodia-filopodia contact sites. (**C**) Quantitation of the co-localization of the components of the nectin and cadherin systems with ZO-1 in ZO-1-knockdown neurons. The data are presented as mean plus SEM (error bars) for each sample (n = 30 for nectin-1, afadin, β-catenin and αN-catenin; n = 40 for nectin-3; n = 20 for N-cadherin) *, p value < 0.05; and **, p value < 0.01 (Student’s t-test) (AU) arbitrary unit. It is noted that the immunofluorescence signals for the components of the nectin and cadherin systems were still observed at some dendritic filopodia-filopodia contact sites in the ZO-1 knockdown neuronal cultures as shown in (**A**) **and **(**B**). We estimated that the transfection efficiency of the siRNA in cultured neurons was about 80%. Thus, ZO-1 might not be knocked-down in some neurons, in which these immunofluorescence signals were observed.

We finally examined the distribution patterns of the components of both the nectin and cadherin systems in the neurons transfected with the EGFP vector and the ZO-1 siRNA. In the ZO-1-kncokdown neurons, the dendritic filopodia-filopodia contacts were reduced and the immunofluorescence signals for nectin-3, afadin, N-cadherin, β-catenin and αN-catenin were hardly observed at any location where they were observed in the control neurons (Figure **7, A1-A3** **and B1-B3, arrowheads, and** Figure **7C**): the average intensities at the dendritic filopodia-filopodia contact sites in the control neurons and the ZO-1-knockdown neurons in arbitrary unit were 140.34 ± 12.62 and 110.62 ± 11.26 for nectin-1, 79.26 ± 7.03 and 47.53 ± 8.63 for nectin-3, 58.77 ± 11.8 and 29.75 ± 4.27 for afadin, 67.14 ± 11.39 and 28.61 ± 5.34 for N-cadherin, 89.37 ± 13.04 and 59.42 ± 1.02 for β-catenin, and 70.31 ± 5.77 and 44.09 ± 7.14 for αN-catenin, respectively. These values are presented as mean ± SEM. The signal for nectin-1 was not apparently changed, but this might be because the amount of nectin-1 in dendrites was not sufficient to detect the significant difference between the control and ZO-1-knockdown neurons. It was confirmed by immunostaining of ZO-1 that ZO-1 was knocked down in the neurons shown in Figure **7** (data not shown). These results indicate that the knockdown of ZO-1 decreases the accumulation of the components of the nectin and cadherin systems at the dendritic filopodia-filopodia contact sites.

## Discussion

It was previously shown by time-lapse fluorescence microscopy of developing hippocampal tissue slices that many fine filopodia dynamically protruded from the dendrites of pyramidal neurons, and that some of them interacted with axons to form synapses [12,13]. The dendritic filopodia were highly motile protrusive structures that protruded and retracted repeatedly. The number of filopodia gradually decreased in close association with an increase in the numbers of stable spine-like structures and resulted in filopodia stabilization and the formation of synapses. However, the behavior or the role of the dendritic filopodia that did not interact with axons was not investigated. By using cultured rat hippocampal neurons, we first confirmed here these earlier observations that many filopodia protrude from the dendrites of hippocampal neurons, and further showed that the dendritic filopodia, which did not make contact with axons, transiently interacted with each other to form dendritic filopodia-filopodia contacts in the early stages of neuronal development. These dendritic filopodia dynamically and repetitively protruded and transiently interacted. Dendritic filopodia, which made contact with other dendritic filopodia, did not become dendritic spines, because dendritic spines were formed only when dendritic filopodia made contact with axons (data not shown). We observed that some dendritic filopodia became the branches of primary dendrites, but we did not observe that the dendritic filopodia-filopodia contacts became the branches of primary dendrites (data not shown). We next showed that the MAGUK family member, ZO-1, which is known to be associated with the nectin and cadherin systems [18,24], was concentrated at the dendritic filopodia-filopodia contact sites and also at the tips of free dendritic filopodia. The overexpression of ZO-1 enhanced the frequency of dendritic filopodial protrusion and their interactions, and stabilized these interactions. In contrast, the knockdown of ZO-1 showed the opposite effects; it decreased the frequency of dendritic filopodial protrusion and their interactions. These results indicate that ZO-1 regulates the dynamic and repetitive protrusion of dendritic filopodia and their transient interactions.

We analyzed here the mode of action of ZO-1 in these behaviors of dendritic filopodia. We showed that the overexpression or knockdown of ZO-1 changed the concentration of the components of the nectin and cadherin systems at the dendritic filopodia-filopodia contact sites. We previously showed that nectin-1 and nectin-3 are asymmetrically localized on the presynaptic and postsynaptic sides, respectively, at the mossy fiber-CA3 pyramidal cell synapses in the stratum lucidum of the mouse hippocampus [28], and that in rat cultured hippocampal neurons in the late stages of neuronal development, nectin-3, but not nectin-1, is distributed in dendrites whereas nectin-1 is mainly distributed in axons, although nectin-3 is also distributed to a lesser extent in axons than in dendrites [29]. Considering our previous observations that the nectin system recruits the cadherin system to the nectin-based cell–cell adhesion sites to form adherens junctions in fibroblasts and epithelial cells [16,25], we proposed from these observations that synapses formed between axons and dendrites are initiated by the trans-interaction between nectin-1 on axons and nectin-3 on dendrites and stabilized by the recruitment of the cadherin system to the nectin-based cell-cell adhesion site. We showed here that in cultured hippocampal neurons in the early stages of neuronal development, not only nectin-3 but also nectin-1 was distributed in dendrites, although the amount of nectin-1 in dendrites was smaller than that in axons. By analogy with the formation of synapses, the dendritic filopodia-filopodia interactions may be initiated by the homophilic and heterophilic trans-interactions of nectin-1 and nectin-3, followed by the recruitment of the cadherin system to the nectin-based contact sites. Different from the relatively stable interactions between axons and dendrites, however, these dendritic filopodia-filopodia interactions were transient and unstable. These different properties between the two types of interaction may be explained by the different combinations of the *trans*-interactions among the nectin family members, which cause the different levels of the recruitment and accumulation of the components of the cadherin system to the nectin-based contact sites [16]. These present and previous results, together with the earlier results that ZO-1 directly interacts with afadin and α-catenin [18,24], suggest that ZO-1 regulates the stabilization of the dendritic filopodia-filopodia interactions by recruiting nectins and N-cadherin through afadin and α-catenin to the contact sites.

We showed here that ZO-1 was localized at the tips of free dendritic filopodia in addition to the dendritic filopodia-filopodia contact sites and that the overexpression or knockdown of ZO-1 changed the frequency of dendritic filopodial protrusion. The molecular mechanisms underlying this localization and the role of ZO-1 remain unknown. However, because many extracellular signaling molecules, such as neurotrophins, are known to induce the formation of dendritic filopodia [31], this localization of ZO-1 may be regulated by these extracellular signaling molecules. Because ZO-1 binds not only to the Cdc42 GEF, Tuba [17,19], but also to afadin and α-catenin [18,24], ZO-1 may function upstream and/or downstream of the Cdc42 signaling pathway in cooperation with afadin and α-catenin. Retraction of extending dendritic filopodia before they interact may be regulated by the inactivation of the ZO-1-Cdc42 signaling pathway, and retraction of dendritic filopodia after they interact may be regulated by both detachment of interacting dendritic filopodia and the inactivation of the ZO-1-Cdc42 signaling pathway.

We showed here that the dynamic and repetitive protrusion of dendritic filopodia and their transient interactions, which were regulated by ZO-1, affected dendrite morphogenesis in cultured neurons. We previously showed that the expression of a chimeric molecule, in which the extracellular region of nectin-1 was fused with the intracellular region of nectin-3 (nectin-1(exo)-nectin-3(intra)), in cultured rat hippocampal neurons caused the ectopic distribution of this chimeric molecule to the dendrites and aberrantly induced the stable dendro-dendritic interactions mediated by endogenous nectin-3 and the exogenous chimera [29]. In addition, expression of this chimeric molecule induced abnormal dendrite morphology. However, the dendrite morphology induced by the overexpression of ZO-1 shown here was different from that induced by the expression of nectin-1(exo)-nectin-3(intra). These results also support the conclusion that the dendritic filopodia-filopodia interactions observed here play a role in dendrite morphogenesis in the early stages of neuronal development, but further indicate that their interaction alone is not sufficient. ZO-1 was localized at the tips of free dendritic filopodia and the growth cones of dendrites and axons in addition to dendritic filopodia-filopodia contact sites. ZO-1 at these other sites may play another role in the extension of dendrites and axons, which may cause the morphological differences between the ZO-1-overexpressing neurons and the nectin-1(exo)-nectin-3(intra)-expressing neurons.

Many mechanisms have been proposed to be involved in dendrite arborization [6]. We showed here another novel mechanism by which ZO-1 influenced dendrite morphogenesis cooperatively with the nectin and cadherin systems by regulating the dynamic and repetitive protrusion of dendritic filopodia and their transient interactions. The most characteristic feature of the abnormal dendrite morphology in the ZO-1-knockdown neurons was that, different from radial extension in the control and ZO-1-overexpressing neurons, the shafts of primary dendrites did not radially extend from the cell body but rather they were fasciculated. These phenotypes in the ZO-1-knockdown neurons were accompanied with the reduction of dendritic filopodia-filopodia contacts. The exact mechanism of the dynamic behaviors of dendritic filopodia in the determination of dendrite morphology remains unknown, but the present results, together with the earlier observations that the Down’s syndrome cell adhesion molecule or proto-cadherins are implicated in dendrite self-avoidance [8-10], suggest that dendritic filopodia dynamically and repetitively protruding from dendrites serve as sensors for dendrite self-avoidance by interacting with each other in the early stages of neuronal development.

## Materials and Methods

### Animal research

Animal experiments were performed in strict accordance with the guidelines of each institution and approved by the administrative panel on laboratory animal care of Kobe University. The protocol was approved by the Committee on the Ethics of Animal Experiments of Kobe University Graduate School of Medicine (Permit Number: P090401). All efforts were made to minimize suffering.

### Cell culture

Rat hippocampal neurons were prepared from embryonic day 18 rat embryos and cultured as described [29] with some modifications. In brief, hippocampi were dissociated by trypsinization and trituration and the dissociated cells were plated at 7.5–15 × 10^3^ cells/cm^2^ on poly-L-lysine–coated glass coverslips. Cultures were maintained in Neurobasal Medium (Invitrogen) with 2% B27 supplement (Invitrogen). The medium was changed every week.

### Transfection

Cultured neurons were transfected with DNA constructs (pCMV-HA-ZO-1 and pCA-EGFP) using an electroporation device (Nucleofector 1; Lonza) on 0 DIV. The generation of the pCMV-HA-ZO-1 DNA construct was described previously [32]. For transfection, the neurons were suspended at 2.5–3.0 × 10^5^ cells/transfection in 100 µl of Amaxa Nucleofector solution and electroporated with 3 µg of DNA constructs, or the neurons were transfected with Effectene (Qiagen). To knock down ZO-1, the neurons were transfected with Stealth Select RNAi siRNA (Invitrogen) for ZO-1 using Lipofectamine RNAiMAX™ (Invitrogen). The siRNA sequence targeting rat ZO-1 corresponded to nucleotides 2,576-2,600 of the rat ZO-1 coding sequence (GACTCCACCGGAGTCTGCTATTACA). In the rescue experiments, the neurons were co-transfected with Stealth Select RNAi siRNA (Invitrogen), pCA-EGFP and pCMV-HA-ZO-1 using siGENE reagents (Promega). In these experiments, pCA-EGFP and pCMV-HA-ZO-1 plasmids were mixed at a mass ratio of 1:2.

### Abs and other materials for immunostaining

The following Abs were obtained from commercial sources: mouse anti-Tau-1 mAb was from Merck Millipore; mouse anti-MAP2 mAb, rabbit anti-β-catenin polyclonal Ab (pAb) and rabbit anti-l-afadin pAb were from SIGMA-Aldrich; mouse anti-ZO-1 mAb, rabbit anti-ZO-1 pAb were from Invitrogen; rat anti-HA mAb was from Roche Applied Science; rabbit anti-GFP pAb was from MBL; rat anti-GFP mAb was from Nacalai Tesque; and rabbit anti-N-cadherin pAb was from Takara Bio. The rabbit anti-nectin-1 pAb and the rabbit anti-nectin-3 pAb were prepared as described [29]. The rat anti-αN-catenin mAb prepared previously [33] was donated from Dr. M. Takeichi (RIKEN CDB). Horseradish peroxidase-conjugated secondary Abs and fluorophore-conjugated secondary Abs were purchased from GE Healthcare, and Merck Millipore, respectively. Alexa Fluor 488 phalloidin, Zenon Alexa Fluor 488 Mouse IgG_2a_ Labeling Kit and Zenon Alexa Fluor 488 Mouse IgG_1_ Labeling Kit were purchased from Invitrogen.

### Immunofluorescence microscopy

Cultured neurons were fixed in HBSS containing 2% or 4% paraformaldehyde at 37°C for 30 min or cold methanol at –20°C for 20 min, then rinsed three times with Tris-buffered saline containing 0.005% Tween-20 (TBS-T) for 2 min, and permeabilized with TBS-T containing 0.25% Triton X-100 at room temperature for 5 min. After being blocked in TBS-T containing 4% goat serum at 37°C for 30 min, the neurons were incubated in TBS-T containing 4% goat serum or Can-get-signal-immunostaining-solution (Toyobo) containing Abs for 75–120 min. The samples were washed three times with TBS-T for 5 min and incubated in TBS-T containing 4% goat serum with the fluorescent secondary Abs for 45 min. The samples were then washed three times with TBS-T for 5 min, mounted with Fluorosave reagent (Merck Millipore), and analyzed on a confocal microscope system (LSM710; Carl Zeiss MicroImaging Inc.).

### Time-lapse imaging

Time-lapse imaging was performed using the LCV110 incubator microscope system (Olympus) equipped with an EMCCD camera (ImagEM, Hamamatsu Photonics) in a combination with a spinning disc laser scan system (CSU-X1, Yokogawa) and imaging software (Metamorph, Molecular Devices). Before experiments, the dissociated cultured hippocampal neurons were plated onto poly-L-lysine coated thin-bottom plastic dishes (Ibidi) and observed with a 20 × NA0.75 lens.

### Image acquisition and statistical analysis

Images of cultured hippocampal neurons were obtained with a confocal microscope system (LSM710; Carl Zeiss MicroImaging Inc.) equipped with a 63× NA 1.4 or a 40× NA 1.3 lens using ZEN LSM710 software (Carl Zeiss MicroImaging Inc.), and their morphology was analyzed with the same software and ImageJ software. For the quantification of dendrite morphology, the number of the shafts of primary dendrites was first counted. The primary dendrites shorter than the diameter of the cell body in length were not counted. The dendrite processes were then manually traced to measure their length using ZEN software; the three longest primary dendrites were chosen for this measurement. The number of the branches protruding from these primary dendrites was also manually counted. The density of filopodia extending from the shafts of the primary dendrites was manually counted; the number of dendritic filopodia extending from the shafts of the primary dendrites within a radius of 50-µm from the center of the cell body was divided by the length of the shafts of the primary dendrites. To obtain the angles of the shafts of each primary dendrite, a circle with a radius of 60-µm on each image was superimposed with the center of the circle placed on the cell body, then the crossing points were plotted, and the radian was measured between points by Sholl analysis with slight modifications. For statistical analysis, parametric analyses were performed with the unpaired Student’s t-test for comparisons of two groups, or one-way ANOVA followed by Tukey-Kramer’s post-hoc test for comparisons of more than two groups. Nonparametric analyses were performed with the Mann-Whitney U test for comparisons of two groups or the Kruskal-Wallis test followed by Steel-Dwass’s post-hoc test for comparisons of more than two groups. In general, several neurons were randomly chosen at least three times from the culture plates for each assay. Neurons on 7 DIV were used for these analyses.

### Western blotting

Hippocampal neurons were cultured on 6-cm poly-L-lysine–coated cell culture dishes and then homogenized on 9 DIV in RIPA buffer (20 mM Tris-HCl, pH 7.5, 1% NP-40, 0.5% sodium deoxycholate, 0.1% SDS, 10% glycerol, 137 mM NaCl, 1 mM CaCl_2_, 1 mM MgCl_2_, 50 mM NaF, 1 mM Na _3_VO_4_, 10 µg/ml leupeptin, 2 µg/ml aprotinin, and a protease inhibitor cocktail (SIGMA-Aldrich)). The protein concentrations of the homogenates were determined using the DC protein assay kit (BIO RAD), and equal amounts of total protein were applied to each lane of SDS-polyacrylamide gels after mixing with an SDS sample buffer (60 mM Tris-HCl, pH 6.7, 3% SDS, 2% 2-mercaptoethanol and 5% glycerol). After separation by SDS-PAGE, the proteins were transferred to poly(vinylidene fluoride) membranes. The membranes were blocked with 5% skim milk at room temperature for 1 h and then incubated with the anti-ZO-1 mAb, the anti-HA mAb or the anti-actin mAb (Merck Millipore) in Can Get Signal solution (Toyobo) at 4°C overnight. The membranes were washed with TBS containing 0.05% Tween-20 and incubated for 1 h in the horseradish peroxidase-conjugated goat secondary Abs (GE Healthcare), and the bands were detected with ECL Plus Substrate (GE Healthcare) or Immobilon (Millipore) using an LAS-4000 mini system (Fuji Film). The signals on the membranes were detected using the LAS-4000, and the intensity of each protein band was analyzed using ImageJ software.

## Supporting Information

Figure S1 **Dendritic filopodia-axons interactions in cultured hippocampal neurons on 14 DIV.** Cultured hippocampal neurons on 14 DIV were triple-stained for F-actin, MAP2 and either β-catenin or ZO-1. (**A**) F-actin, β-catenin and MAP2; (**B**) F-actin, ZO-1 and MAP2. Upper rows, low magnification images; lower rows, high magnification images of the boxed areas in the upper rows. Bars, upper rows 10 µm; lower rows 2.5 µm. Arrowheads indicate dendritic filopodia-axons contact sites.
(TIF)

Figure S2 **Western blots for ZO-1-overexpressing neurons and ZO-1-knockdown neurons.** (**A**) HA-tagged ZO-1-overexpressing neurons. The lysates were harvested on 9 DIV from the neurons transfected with the empty vector or the HA-tagged ZO-1 vector on 0 DIV; (**B**) ZO-1-knockdown neurons. The lysates were harvested on 9 DIV from the neurons transfected with the control siRNA or the ZO-1 siRNA on 3 DIV. Actin was used as the control.
(TIF)

Figure S3 **Restoration of the dendrite morphology of the ZO-1 knockdown neurons by re-expression of ZO-1.** (**A**) Restoration of the dendrite morphology of ZO-1 knockdown neurons by re-expression of the siRNA-resistant ZO-1. Cultured hippocampal neurons were transfected with the ZO-1 siRNA, the EGFP expression vector and either the empty (MOCK) or siRNA-resistant ZO-1vectors on 0 DIV, and triple-stained for EGFP, ZO-1 and MAP2 on 7 DIV. **Upper rows**, ZO-1-knockdown neurons expressing EGFP and the empty vector; **lower rows**, ZO-1-knockdown neurons expressing EGFP and the siRNA-resistant ZO-1 vector. **Bars**, 10 µm. (**B**) Statistical analysis of the dendrite morphology of the neurons. **Control**, the control siRNA and the EGFP vector; **ZO-1 KD**, the ZO-1 siRNA and the EGFP vector; **ZO-1 KD + HA-ZO-1**, the ZO-1 siRNA, the EGFP vector and siRNA-resistant ZO-1; **ZO-1 KD + MOCK**, the ZO-1 siRNA, the EGFP vector and the empty vector. The average number of the shafts of the primary dendrites, the average length of the shafts of the primary dendrites, the average number of the shafts of the branches of the primary dendrites, the average density of the dendritic filopodia protruding from the shafts of the primary dendrites and the angles of the shafts of each primary dendrite in the neurons transfected with the indicated combinations of the siRNAs and the expression vectors on 0 DIV were measured on 7 DIV. The data are presented as mean plus SEM (error bars) for each sample (n = 8 for the number of the shafts of the primary dendrites (**Number of primary dendrite shafts**); n = 24 for the length of the shafts of the primary dendrites (**Length of primary dendrite shafts**); n = 24 for the number of the shafts of the branches of the primary dendrites (**Number of branch shafts**); n = 48 for the density of dendritic filopodia protruding from the shafts of the primary dendrites (**Density of dendritic filopodia**); and n = 48 for the angles of the shafts of the primary dendrites (**Angle of primary dendrite shafts**)). Statistical analyses of the dendrite morphology of except for that of the angles of each dendrite were performed using one-way ANOVA followed by Tukey-Kramer’s post-hoc test. Statistical analysis of the angles of the shafts of each primary dendrite was performed using Kruskal–Wallis test followed by Steel-Dwass’s post-hoc test. *, p value < 0.05; and **, p value < 0.01 (one-way ANOVA followed by Tukey-Kramer’s post-hoc test) and ***, p value < 0.01 (Kruskal–Wallis test followed by Steel-Dwass’s post-hoc test). The average number of the shafts of the primary dendrites was 5.5 ± 0.38 in **Control**, that was 4.75 ± 0.77 in **ZO-1 KD**, that was 4.5 ± 0.46 in **ZO-1 KD + HA-ZO-1**, and that was 5.25 ± 0.56 in **ZO-1 KD + MOCK**. The average length of the shafts of the primary dendrites protruding from the cell body was 60.82 ± 4.50 µm in **Control**, that was 72.59 ± 7.55 µm in **ZO-1 KD**, that was 62.95 ± 10.80 µm in **ZO-1 KD + HA-ZO-1**, and that was 83.04 ± 10.10 µm in **ZO-1 KD + MOCK**. The average number of the shafts of the branches of the primary dendrites was 2.33 ± 0.39 in **Control**, that was 1.17 ± 0.27 in **ZO-1 KD**, that was 1.83 ± 0.31 in **ZO-1 KD + HA-ZO-1**, and that was 1.38 ± 0.22 in **ZO-1 KD + MOCK**. The average density of the dendritic filopodia protruding from the primary dendrites was 1.22 ± 0.09 per 10 µm in **Control**, that was 0.72 ± 0.08 per 10 µm in **ZO-1 KD**, that was 1.04 ± 0.11 per 10 µm in **ZO-1 KD + HA-ZO-1**, and that was 0.72 ± 0.07 per 10 µm in **ZO-1 KD + MOCK**. The median angle of the shafts of the primary dendrites was 45.90 degree in **Control**, that was 27.40 degree, that was 45.00 degree in **ZO-1 KD**, and that was 28.60 degree in **ZO-1 KD + MOCK**. These values were estimated by the signals for EGFP and MAP2. The values of the length of the shafts of the primary dendrites and the density of the dendritic filopodia protruding from the shafts of the primary dendrites the angles of the shafts of each primary dendrite in (**B**) were different from those in the Figure **4C**. These differences were due to different assay conditions. It is noted that we attempted to express HA-tagged ZO-1 in the ZO-1-knockdown neurons under various assay conditions to adjust its expression level similar to that in the wild-type neurons, because both overexpression and knockdown of ZO-1 caused different morphological changes of dendrites. However, in the ZO-1-knockdown neurons we could not perfectly restore the ZO-1 expression level to its endogenous level in the wild-type neurons. Therefore, the reason why the transfection of this vector did not increase the numbers of the shafts of primary dendrites and their branches or the length of the shafts of the primary dendrites might be due to the insufficient expression level of HA-tagged ZO-1.
(TIF)

Video S1 **Transient dendritic filopodia-filopodia interactions in cultured hippocampal neurons.** Time-lapse movies of cultured hippocampal neurons. Images were captured every 30 min from 3 to 7 DIV.
(MOV)

Video S2 **Dendritic filopodia protrusion, their interactions and dendrite morphology in neurons (Control for video 3).** Time-lapse movies of control neurons. Images of a neuron expressing both EGFP and the empty vector were captured every 30 min from 5 to 6 DIV.
(MOV)

Video S3 **Enhancement of dendritic filopodia protrusion, stabilization of their interactions and changes in dendrite morphology in ZO-1-overexpressing neurons.** Time-lapse movies of HA-tagged ZO-1-overexpressing neurons. Images of a neuron expressing EGFP and HA-tagged ZO-1 were captured every 30 min from 5 to 6 DIV.
(MOV)

Video S4 **Dendritic filopodia protrusion and dendrite morphology in neurons (Control for video 5).** Time-lapse movies of control neurons. Images of a neuron expressing EGFP and the control siRNA were captured every 30 min from 5 to 6 DIV.
(MOV)

Video S5 **Enhanced protrusion of dendritic filopodia and changes in dendrite morphology in ZO-1-knockdown neurons.** Time-lapse movies of ZO-1-knockdown neurons. Images of a neuron expressing EGFP and the ZO-1 siRNA were captured every 30 min from 5 to 6 DIV.
(MOV)
