# Supplementary figures and images for: Regulation of Dendritic Filopodial Interactions by ZO-1 and Implications for Dendrite Morphogenesis
Source: PLoS One. 2013 Oct 2;8(10):e76201. doi: 10.1371/journal.pone.0076201 (PMC3788765; doi:10.1371/journal.pone.0076201)

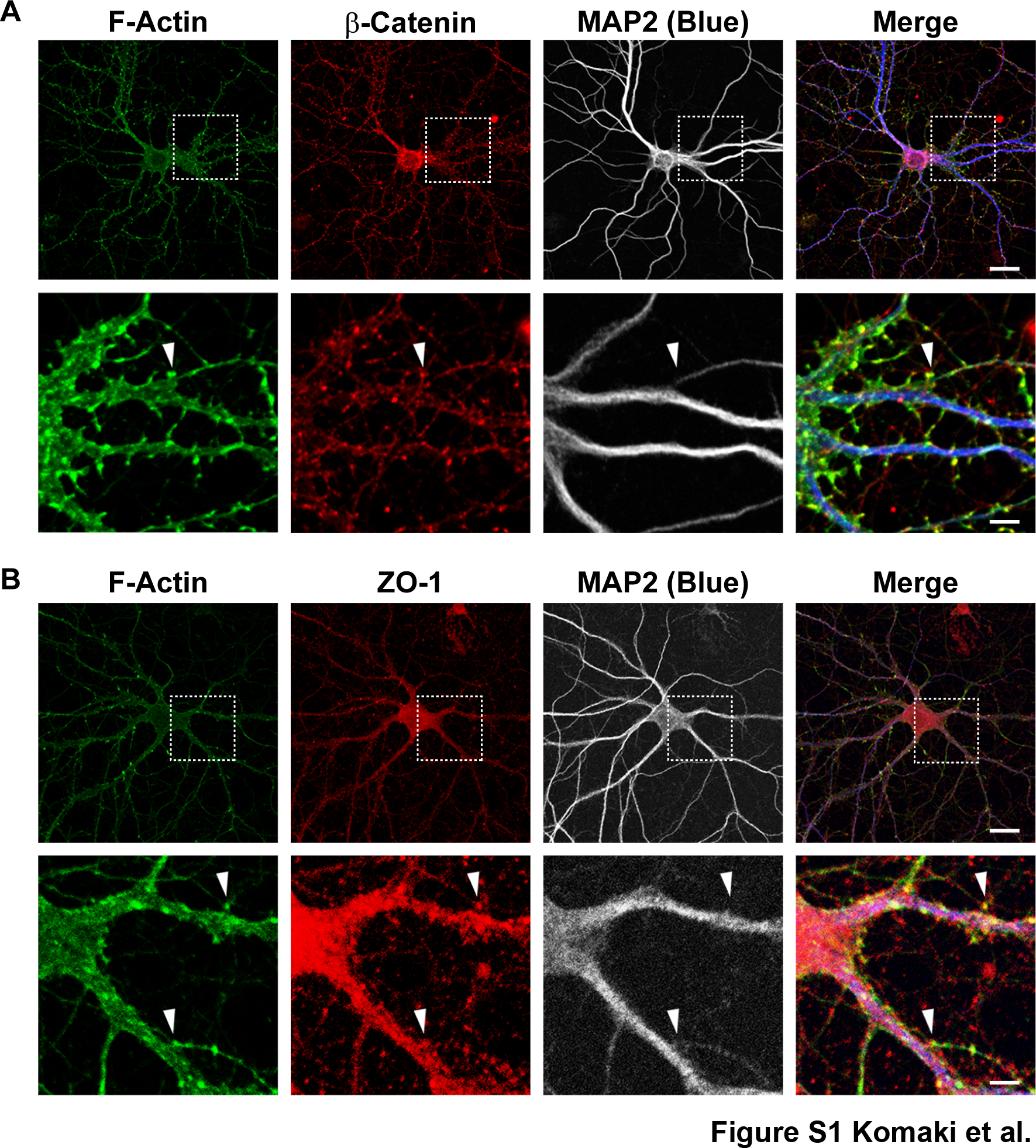

Supplement: Figure S1 — Dendritic filopodia-axons interactions in cultured hippocampal neurons on 14 DIV. Cultured hippocampal neurons on 14 DIV were triple-stained for F-actin, MAP2 and either β-catenin or ZO-1. (A) F-actin, β-catenin and MAP2; (B) F-actin, ZO-1 and MAP2. Upper rows, low magnification images; lower rows, high magnification images of the boxed areas in the upper rows. Bars, upper rows 10 µm; lower rows 2.5 µm. Arrowheads indicate dendritic filopodia-axons contact sites. (TIF) [file pone.0076201.s001.tif]

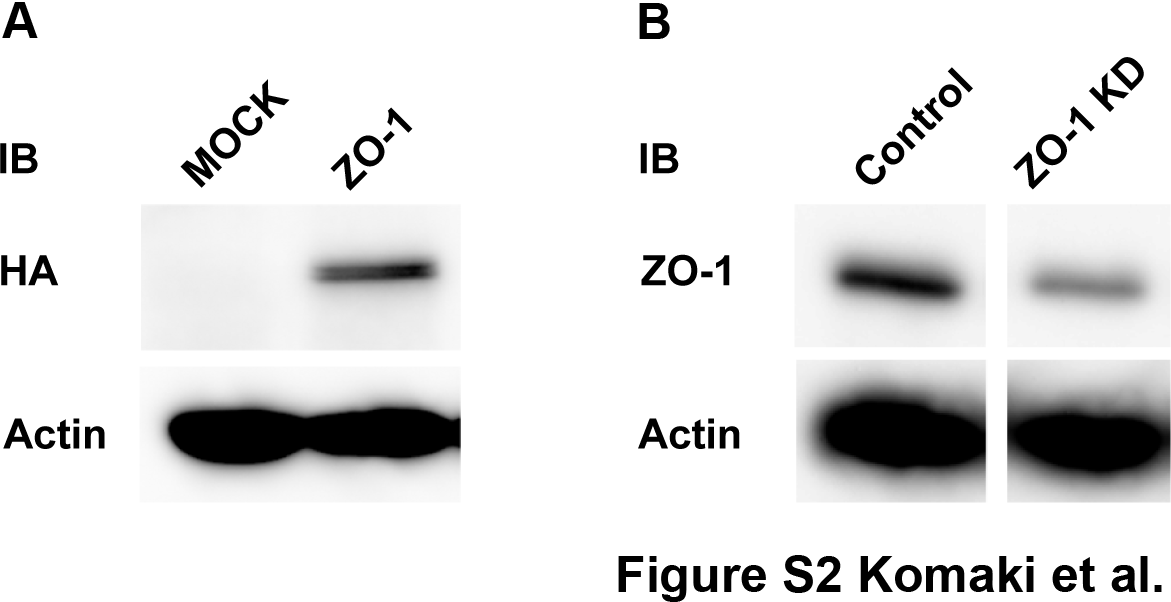

Supplement: Figure S2 — Western blots for ZO-1-overexpressing neurons and ZO-1-knockdown neurons. (A) HA-tagged ZO-1-overexpressing neurons. The lysates were harvested on 9 DIV from the neurons transfected with the empty vector or the HA-tagged ZO-1 vector on 0 DIV; (B) ZO-1-knockdown neurons. The lysates were harvested on 9 DIV from the neurons transfected with the control siRNA or the ZO-1 siRNA on 3 DIV. Actin was used as the control. (TIF) [file pone.0076201.s002.tif]

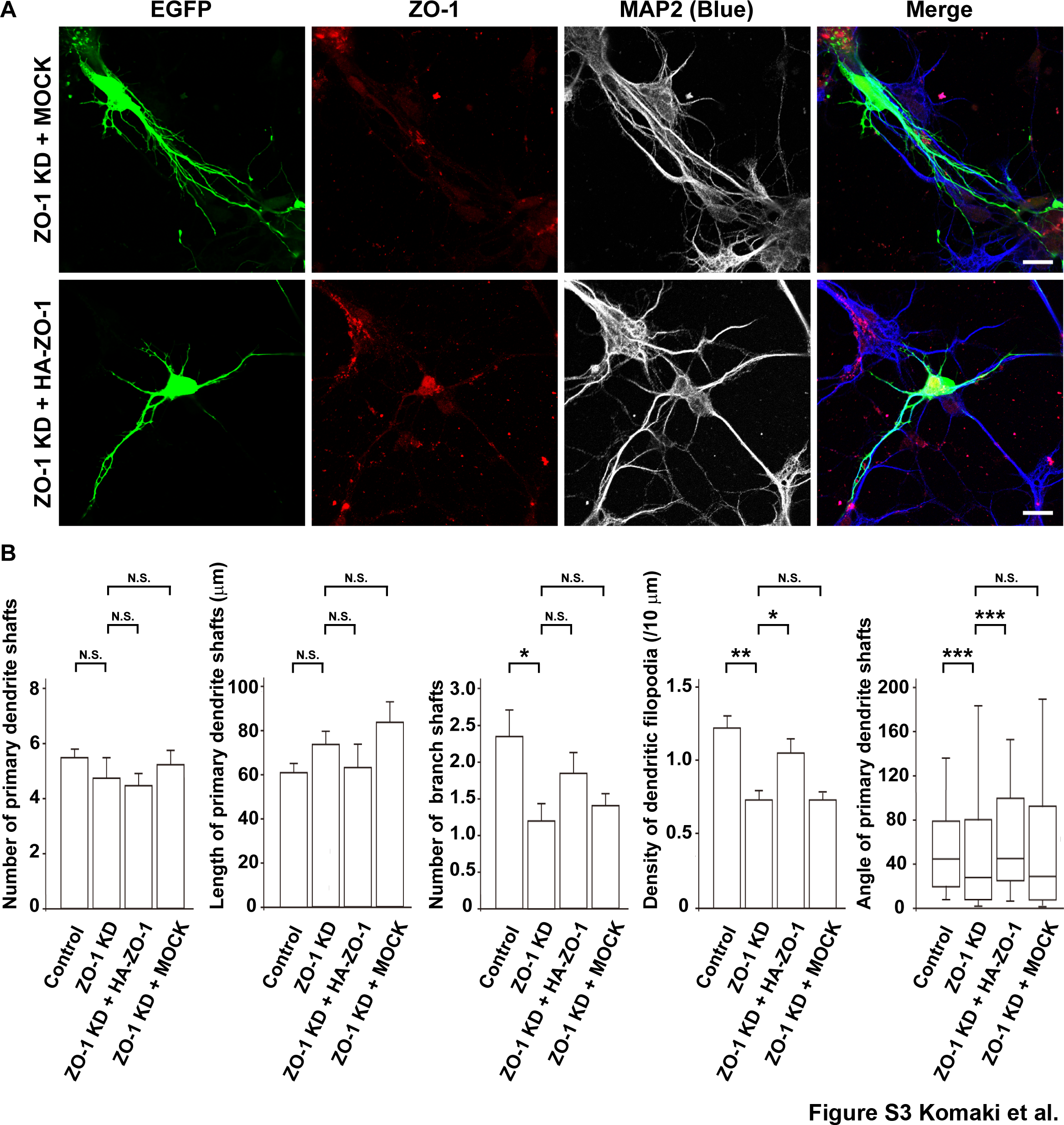

Supplement: Figure S3 — Restoration of the dendrite morphology of the ZO-1 knockdown neurons by re-expression of ZO-1. (A) Restoration of the dendrite morphology of ZO-1 knockdown neurons by re-expression of the siRNA-resistant ZO-1. Cultured hippocampal neurons were transfected with the ZO-1 siRNA, the EGFP expression vector and either the empty (MOCK) or siRNA-resistant ZO-1vectors on 0 DIV, and triple-stained for EGFP, ZO-1 and MAP2 on 7 DIV. Upper rows, ZO-1-knockdown neurons expressing EGFP and the empty vector; lower rows, ZO-1-knockdown neurons expressing EGFP and the siRNA-resistant ZO-1 vector. Bars, 10 µm. (B) Statistical analysis of the dendrite morphology of the neurons. Control, the control siRNA and the EGFP vector; ZO-1 KD, the ZO-1 siRNA and the EGFP vector; ZO-1 KD + HA-ZO-1, the ZO-1 siRNA, the EGFP vector and siRNA-resistant ZO-1; ZO-1 KD + MOCK, the ZO-1 siRNA, the EGFP vector and the empty vector. The average number of the shafts of the primary dendrites, the average length of the shafts of the primary dendrites, the average number of the shafts of the branches of the primary dendrites, the average density of the dendritic filopodia protruding from the shafts of the primary dendrites and the angles of the shafts of each primary dendrite in the neurons transfected with the indicated combinations of the siRNAs and the expression vectors on 0 DIV were measured on 7 DIV. The data are presented as mean plus SEM (error bars) for each sample (n = 8 for the number of the shafts of the primary dendrites (Number of primary dendrite shafts); n = 24 for the length of the shafts of the primary dendrites (Length of primary dendrite shafts); n = 24 for the number of the shafts of the branches of the primary dendrites (Number of branch shafts); n = 48 for the density of dendritic filopodia protruding from the shafts of the primary dendrites (Density of dendritic filopodia); and n = 48 for the angles of the shafts of the primary dendrites (Angle of primary dendrite shafts)). Statistica [file pone.0076201.s003.tif]
